# Association between nonsteroidal anti-inflammatory drugs use and risk of central nervous system tumors: a dose-response meta analysis

**DOI:** 10.18632/oncotarget.21829

**Published:** 2017-10-11

**Authors:** Tao Zhang, Xiaowen Yang, Pei Liu, Jianrui Zhou, Jie Luo, Hui Wang, Anrong Li, Yi Zhou

**Affiliations:** ^1^ Department of Neurosurgery, Taihe Hospital, Hubei University of Medicine, Shiyan, Hubei, 442000, China; ^2^ Department of Clinical Laboratory, Taihe Hospital, Hubei University of Medicine, Shiyan, Hubei, 442000, China; ^3^ Department of Dermatology, Taihe Hospital, Hubei University of Medicine, Shiyan, Hubei, 442000, China; ^4^ Department of Rehabilitation Medicine, Taihe Hospital, Hubei University of Medicine, Shiyan, Hubei, 442000, China

**Keywords:** central nervous system tumors, nonsteroidal anti-inflammatory drugs, dose-response relationship, meta analysis

## Abstract

Although studies have examined the association between nonsteroidal anti-inflammatory drugs (NSAIDs) use and central nervous system (CNS) tumors risk, the results are inconclusive. Here, we conducted a dose-response meta-analysis in order to investigate the correlation between NSAIDs use and CNS tumors risk. Up to July 2017, 12 studies were included in current meta-analysis. NSAIDs use was significantly associated with a lower risk of CNS tumors. Furthermore, non-aspirin NSAIDs or aspirin use are significantly associated with a lower risk of CNS tumors. Additionally, NSAIDs use was associated with significantly a lower risk of glioma, glioblastoma but not meningioma. Subgroup analysis showed consistent findings. Furthermore, a significant dose-response relationship was observed between NSAIDs use and CNS tumors risk. Increasing cumulative 100 defined daily dose of NSAIDs use was associated with a 5% decrement of CNS tumors risk, increasing NSAIDs or non-aspirin NSAIDs or aspirin use (per 3 prescriptions increment) was associated with a 7%, 7%, 10% decrement of CNS tumors risk, increasing per 2 year of duration of NSAIDs or non-aspirin NSAIDs or aspirin use was associated with a 6%, 8%, 6% decrement of CNS tumors risk. Considering these promising results, NSAIDs use might provide helpful for reducing CNS tumors risk. Large sample size and different ethnic population are warranted to validate this association.

## INTRODUCTION

Central nervous system (CNS) tumors are the second leading cause of death from neurological diseases worldwide, and costs on patients, caregivers and society [1]. Survival chances have improved gradually over the last 30 years but remain poor compared to many other cancers, and 30% glioma survived to one year and 15% glioma survived to five years of patients after diagnosis in adults [2, 3]. These data reveal the poor prognosis of CNS tumors, and thus to prevent the occurrence of CNS tumors is essential. The etiology of CNS tumors involves both genetic and environmental factors. Compared with many other cancers, there are only a few identified risk factors for glioma, including increasing age, male, rare genetic syndrome, and high levels of ionizing radiation [4]. Meanwhile, previous studies investigating have showed nonsteroidal anti-inflammatory drugs (NSAIDs) have a chemopreventive potential in the CNS tumors *in vitro* and *in vivo* [5].

NSAIDs are a non-steroidal anti-inflammatory drugs, including aspirin, acetaminophen, indomethacin, naproxen, diclofenac, ibuprofen, nimesulide, rofecoxib and celecoxib. The main function of NSAIDs is anti-inflammatory, antirheumatic, relieve pain and anticoagulation [6, 7]. At present, NSAIDs is one of the most widely used drugs in the world. About 30 million people use it every day around the world. As people using NSAIDs increases, clinicians, pharmacists, patients, and society and governments pay more attention to the safety of these drugs.

Previous studies have examined the relationship between NSAIDs use and risk of colorectal cancer [8], stomach cancer [9], prostate cancer [10] and breast cancer [11], have found that NSAIDs use is significantly reduce cancer risk. Even though some studies supported NSAIDs use significantly decrease the risk of CNS tumors [12–23]. However, the result remains controversial. Additionally, no study to quantitative assessed NSAIDs use in relation to CNS tumors. Thus, we performed this dose-response meta-analysis to clarify and quantitative assessed the correlation between NSAIDs use and CNS tumors risk.

## RESULTS

### Literature search results

**Figure 1:**
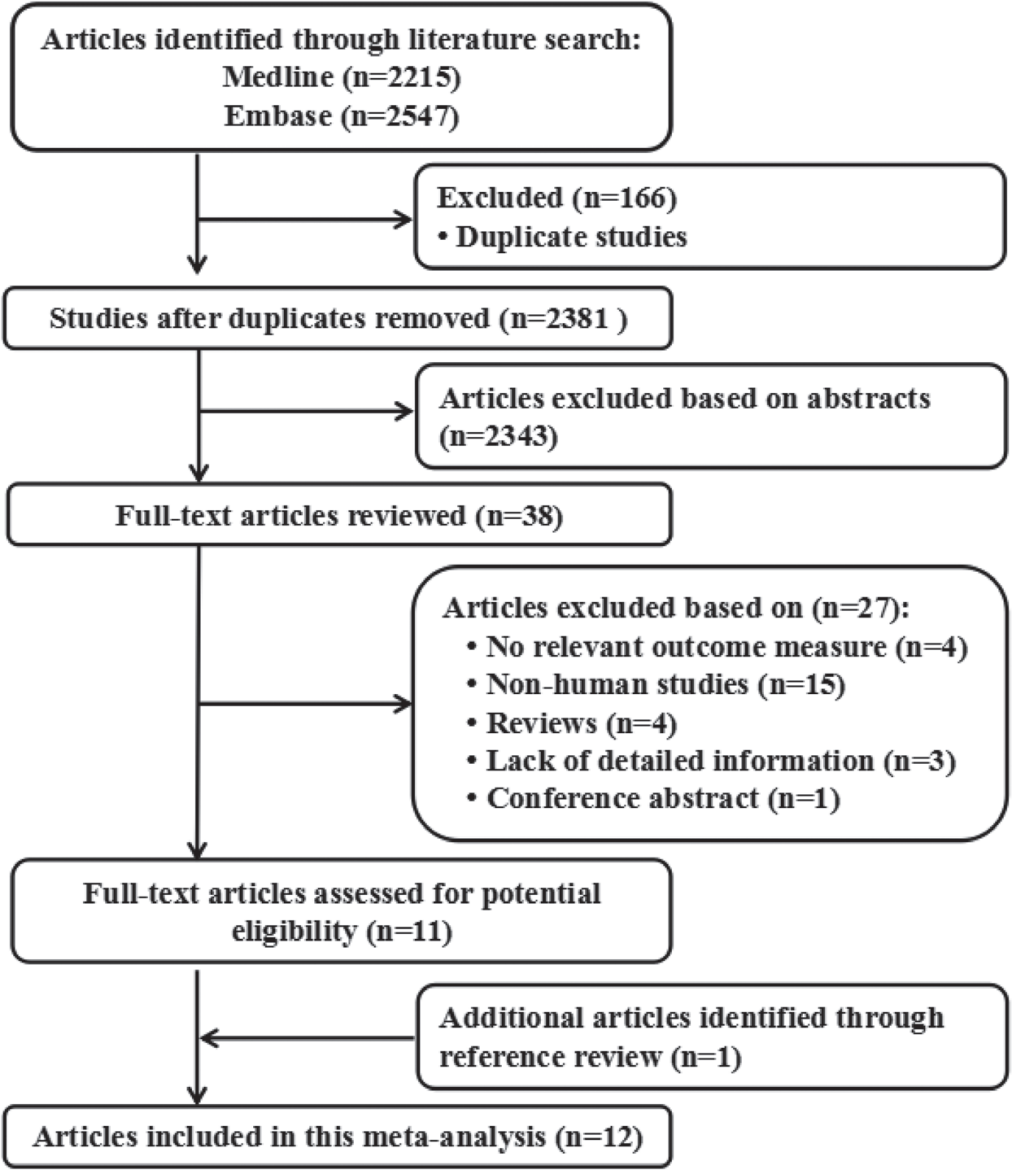
Flow diagram of the study selection process

Figure 1 shows literature research and selection. A total of 2215 studies from PubMed and 2547 studies from Embase. After exclusion of duplicates and studies that did not fulfill the inclusion criteria, 12 studies were chosen, and the data were extracted. These studies were published update to July 2017.

### Study characteristics

The characteristics of the included studies of NSAIDs use and CNS tumors risk are shown in the Table 1 and 2. Results in different subgroups were treated as two separate reports. Finally, thirty-three independent reports from twelve studies investigated the association between NSAIDs use and CNS tumors risk. Among the selected reports, twenty-two reports investigated the association between non-aspirin NSAIDs use and CNS tumors risk, eleven reports investigated the association between aspirin use and CNS tumors risk. A total of 667085 participants with 19394 incident cases from three countries were included in this meta-analysis.

**Table 1:** Characteristics of participants in included studies of nonsteroidal anti-inflammatory drugs using in relation to risk of central nervous system tumor

Author(year)	Study design	Country	Sex of population	Age at baseline (years)	No of participants	Endpoints (cases)	Quality score
Bannon et al. (2013)	Case-control	United Kingdom	Mix	58.0	47730	Brain tumours (5052) Gliomas (2313) Meningiomas (861)	7
Gaist et al. (2013)	Case-control	Denmark	Mix	20–85	21536	Gliomas (2688)	7
Sivak-Sears et al. (2004)	Case-control	USA	Mix	51–71.5	637	Glioblastoma (236)	7
Scheurer et al. (2008)	Case-control	USA	Mix	50	925	Gliomas (325)	6
Scheurer et al. (2011)	Case-control	USA	Mix	53.5	2873	Gliomas (1339)	7
Ferris et al. (2012)	Case-control	USA	Mix	57.4	917	Gliomas (517)	6
Seliger et al. (2015)	Case-control	United Kingdom	Mix	55.5	22055	Gliomas (2005)	7
Seliger et al. (2016)	Case-control	United Kingdom	Mix	55.3	27159	Gliomas (2469)	7
Friis et al. (2003)	Cohort	Denmark	Mix	70.0	29470	Brain tumours (193)	7
Sørensen et al. (2003)	Cohort	USA	Mix	47.2	172057	Brain tumours (170)	8
Daugherty et al. (2011)	Cohort	USA	Mix	63.4	302767	Gliomas (674) Glioblastoma (521)	7
Cook et al. (2005)	Randomizedclinical trial	USA	Female	54.6	39876	Brain tumours (31)	6

**Table 2:** Outcomes and covariates of included studies of nonsteroidal anti-inflammatory drugs using in relation to risk of central nervous system tumor

Author (year)	Endpoints	Data source	Category and relative risk (95% CI)	Covariates in fully adjusted model
Bannon et al. (2013)	Brain tumours (5052) Gliomas (2313) Meningiomas (861)	Population-based	**Non-aspirin NSAIDs** **Gliomas** 0 DDDs, 1.0 (reference); > 0- < 28, 1.00 (0.85, 1.16); > 28- < 65, 0.99 (0.85, 1.16); > 65- < 212, 1.21 (1.04, 1.40); > 212, 0.96 (0.82, 1.1.13) **Meningiomas** 0 DDDs, 1.0 (reference); > 0- < 28, 1.02 (0.79, 1.31); > 28- < 65,1.40 (1.10, 1.77); > 65- < 212, 1.32 (1.04, 1.67); > 212, 1.35 (1.06, 1.71) **Aspirin** **Gliomas** 0 DDDs, 1.0 (reference); > 0- < 224, 1.08 (0.85, 1.38); > 224- < 728, 1.09 (0.86, 1.40); > 728- < 1572, 1.01(0.78,1.31); > 1572, 0.85 (0.65, 1.12) **Meningiomas** 0 DDDs, 1.0 (reference); > 0- < 224, 1.39 (0.98, 1.97); > 224- < 728, 1.29 (0.88, 1.90); > 728- < 1572, 0.88 (0.58,1.32); > 1572, 0.97 (0.64, 1.47)	Adjusted for age,sex, history of osteoarthritis/arthralgia;history of rheumatoid arthritis, history of allergy, history of hormone replacement therapy use.
Gaist et al. (2013)	Gliomas (2688)	Self-administered	**Recent use, years** **Non-aspirin NSAIDs** Never use, 1.0 (reference); > 0- < 2, 1.06 (0.95, 1.17); > 2- < 4, 0.97 (0.60, 1.57); > 5, 1.11 (0.57, 2.17) **Aspirin** Never use, 1.0 (reference); > 0- < 2, 0.96 (0.79, 1.16); > 2- < 4, 0.80 (0.60, 1.06); > 5, 0.80 (0.53, 1.21) **Past use, years** **Non-aspirin NSAIDs** Never use, 1.0 (reference); > 0- < 2, 1.05 (0.95, 1.17); > 2, 0.91 (0.29, 2.83) **Aspirin** Never use, 1.0 (reference); > 0- < 2, 0.84(0.61, 1.15); > 2, 0.47 (0.14, 1.15)	Adjusted for education, diabetes, stroke, allergy, asthma, use of statins, antihistamines, and anti-asthma medication
Sivak-Sears et al. (2004)	Glioblastoma (236)	Population-based	**Recent use, years** **Ibuprofen** Never use, 1.0 (reference); > 0- < 5, 0.32 (0.10, 0.70); > 5, 1.04 (0.60, 1.80) **Naproxen** Never use, 1.0 (reference); > 0- < 3, 0.75 (0.30, 1.70); > 3, 0.50 (0.20, 1.20) **Acetaminophen** Never use, 1.0 (reference); > 0- < 10, 0.72 (0.30, 1.50); > 10, 1.08 (0.60, 2.00) **Aspirin** Never use, 1.0 (reference); > 0- < 10, 0.71 (0.40, 1.20); > 10, 0.66 (0.40, 1.10)	Adjustment for gender, ethnicity, income, and education
Scheurer et al. (2011)	Gliomas (1339)	Population-based	**NSAIDs** Never use, 1.0 (reference); > 0- < 10, 0.55 (0.35, 0.88); > 10, 0.65 (0.39, 1.07)	Adjusted for age, race, sex, education, study series, family history of brain tumors, and history of chickenpox and controlled for all other covariates in the table.
Ferris et al. (2012)	Gliomas (517)	Self-administered	**Recent use, years** **Aspirin** < 6 Months, 1.0 (reference); > 7- < 24, 0.57 (0.30, 1.08); > 25- < 60, 0.64 (0.38, 1.06); > 60, 0.80 (0.51, 1.24) **Ibuprofen** < 6 Months, 1.0 (reference); > 7- < 24, 1.49 (0.40, 5.54); > 25- < 60, 1.06 (0.55, 2.06); > 60, 1.08 (0.64, 1.84) **Naproxen** < 6 Months, 1.0 (reference); > 7- < 24, 3.40 (0.68, 17.34); > 25- < 60, 0.85 (0.23, 2.98); > 60, 0.37 (0.13, 1.11) **All NSAIDs** < 6 Months, 1.0 (reference); > 7- < 24, 0.70 (0.35, 1.38); > 25- < 60, 0.75 (0.46,1.23); > 60- < 120, 0.73 (0.49, 1.08); > 120, 0.62 (0.38, 1.00)	Adjusted for individual statins, NSAIDs, age, race, gender and center
Seliger et al. (2015)	Gliomas (2005)	Population-based	**Number of prescriptions** **NSAIDs** Never use, 1.0 (reference); > 1- < 9, 0.98 (0.89, 1.08); > 10, 1.05 (0.84, 1.33) **Aspirin** Never use, 1.0 (reference); > 1- < 14, 0.78 (0.55, 1.11); > 15, 1.22 (0.76, 1.94)	Adjusted for age, sex, general practice, number of years of active history in the database, and adjusted for BMI and smoking
Seliger et al. (2016)	Gliomas (2469)	Self-administered	**Number of prescriptions** **Aspirin** Never use, 1.0 (reference); > 1- < 9, 0.83(0.60, 1.15); > 10- < 29, 0.80 (0.43, 1.50); > 30, 1.19 (0.66, 2.13) **COX-2 inhibitors** Never use, 1.0 (reference); > 1- < 9, 1.02 (0.92, 1.13); > 10- < 29, 1.01 (0.80, 1.28); > 30, 1.16 (0.76, 1.55) **Ibuprofen** Never use, 1.0 (reference); > 1- < 9, 0.95 (0.86, 1.05); > 10- < 29, 1.03 (0.77, 1.39); > 30, 0.94 (0.60, 1.48) **Naproxen** Never use, 1.0 (reference); > 1- < 9, 0.91 (0.79, 1.05); > 10- < 29, 0.52 (0.28, 0.96); > 30, 1.45 (0.83, 2.52)	Adjusted for age, sex, general practice, and number of years of active history in the database, body mass index, smoking, diabetes, congestive heart failure, and all other medications in this table
Daugherty et al. (2011)	Gliomas (674) Glioblastoma (521)	Population-based	**Number of prescriptions** **Glioma** **Aspirin** Never use, 1.0 (reference); > 0- < 2, 1.21(0.91, 1.61); > 2- < 6, 1.07 (0.70, 1.62); > 7, 1.21 (0.88, 1.65) **Non Aspirin NSAID** Never use, 1.0 (reference); > 0- < 2, 1.06(0.84, 1.33); > 2- < 6, 0.87 (0.53, 1.44); > 7, 0.92 (0.62, 1.36) **Glioblastoma** **Aspirin** Never use, 1.0 (reference); > 0- < 2, 1.30(0.94, 1.80); > 2- < 6, 1.06 (0.65, 1.72); > 7, 1.21 (0.84, 1.75) **Non Aspirin NSAID** Never use, 1.0 (reference); > 0- < 2, 1.03(0.79, 1.34); > 2- < 6, 0.58 (0.30, 1.15); > 7, 0.96 (0.62, 1.49)	Adjusting for sex, race, and history of heart disease using age as time metric

Abbreviations: DDD: defined daily dose.

### NSAIDs use and CNS tumors risk

Thirty-three independent reports from twelve studies investigated the association between NSAIDs use and CNS tumors risk. Compared with no NSAIDs use, NSAIDs use was significantly associated with a lower risk of CNS tumors risk (RR:0.89; 95% CI, 0.81–0.95; *P* = 0.001) (Table 3). Furthermore, NSAIDs use was associated with significantly a lower risk of glioma (RR:0.92; 95% CI, 0.87–0.98; *P* = 0.012) (Table 3), glioblastoma (RR:0.86; 95% CI, 0.73–0.98; *P* < 0.001) (Table 3) but not meningioma (RR:0.73; 95% CI, 0.48–1.12; *P* = 0.149) (Table 3). That may be because there isn't enough data in meningioma and CNS tumors risk.

**Table 3:** Stratified analyses of relative risk of central nervous system tumor risk

Studies groups	No of reports	Relative risk (95% CI)	Heterogeneity		*P* for test
			*P* value	I^2^ (%)	
Total	33	0.89 (0.81–0.95)	0.000	55.8%	0.001
Subgroup analyses for nonsteroidal anti-inflammatory drugs using					
**Type of drugs use**					
Non-aspirin NSAIDs using	22	0.86 (0.78–0.92)	0.001	54.9%	0.002
Aspirin using	11	0.88 (0.79–0.95)	0.014	54.8%	0.006
**Tumour subtype**					
Glioma	18	0.92 (0.87–0.98)	0.000	55.3%	0.012
Meningioma	2	0.73 (0.48–1.12)	0.135	55.3%	0.149
Glioblastoma	7	0.86 (0.73–0.98)	0.177	39.1%	< 0.001
**Study design**					
Case–control	27	0.89 (0.83–0.96)	0.000	60.5%	0.001
Cohort	6	0.93 (0.83–0.99)	0.256	23.7%	0.043
**No of participants**					
≥ 10 000	23	0.94 (0.86–0.99)	0.000	57.6%	0.038
< 10 000	10	0.72 (0.62–0.83)	0.705	0.0%	< 0.001
**No of cases**					
≥1000	21	0.94 (0.87–0.99)	0.000	59.1%	0.041
<1000	12	0.80 (0.68–0.90)	0.051	43.8%	< 0.001
**Study quality**					
Score ≥ 7	30	0.83 (0.73–0.93)	0.000	65.4%	< 0.001
Score < 7	3	0.95 (0.86–1.05)	0.313	0.0%	0.413

### Non-aspirin NSAIDs use and CNS tumors risk

Twenty-two independent reports from ten studies investigated the association between non-aspirin NSAIDs use and CNS tumors risk. Non-aspirin NSAIDs use was significantly associated with a lower risk of CNS tumors risk (RR:0.86; 95% CI, 0.78–0.94; *P* = 0.002) (Table 4). Furthermore, non-aspirin NSAIDs use was associated with significantly a lower risk of glioma (RR:0.94; 95% CI, 0.88–0.99; *P* = 0.042) (Table 4), glioblastoma (RR:0.78; 95% CI, 0.63–0.95; *P* = 0.014) (Table 4) but not meningioma (RR:0.97; 95% CI, 0.64–1.47; *P* = 0.880) (Table 4).

**Table 4:** Associations between non-aspirin NSAIDs using and central nervous system tumor risk in subgroup meta-analyses

Studies groups	No of reports	Relative risk (95% CI)	Heterogeneity		*P* for test
			*P* value	I^2^ (%)	
Total	22	0.86 (0.78–0.92)	0.001	54.9%	0.002
**Tumour subtype**					
Glioma	16	0.94 (0.88–0.99)	0.003	57.0%	0.042
Meningioma	1	0.97 (0.64–1.47)			0.880
Glioblastoma	5	0.78 (0.63–0.95)	0.883	0.0%	0.014
**Study design**					
Case–control	19	0.88 (0.78–0.95)	0.000	60.3%	< 0.001
Cohort	3	0.93 (0.85–0.98)	0.686	0.0%	0.013
**No of participants**					
≥ 10 000	17	0.87 (0.78–0.91)	0.000	66.0%	0.003
< 10 000	5	0.91 (0.85–0.97)	0.155	46.3%	< 0.001
**No of cases**					
≥ 1000	11	0.83 (0.73–0.94)	0.162	24.2%	0.005
< 1000	11	0.90 (0.81–0.98)	0.001	65.8%	0.013
**Study quality**					
Score ≥ 7	20	0.88 (0.78–0.98)	0.002	54.6%	0.007
Score < 7	2	0.74 (0.67–0.82)	0.448	0.0%	< 0.001

### Aspirin use and CNS tumors risk

Eleven independent reports from seven studies investigated the association between aspirin use and CNS tumors risk. Compared with no aspirin use, aspirin use was significantly associated with a lower risk of CNS tumors risk (RR:0.88; 95% CI, 0.79–0.95; *P* = 0.006)(Table 5). Furthermore, aspirin use was associated with significantly a lower risk of glioma(RR:0.86; 95% CI, 0.76–0.95; *P* < 0.001) (Table 5) but not meningioma (RR:0.85; 95% CI, 0.65–1.12; *P* = 0.253) (Table 5) and glioblastoma (RR:0.91; 95% CI, 0.55–1.50; *P* = 0.701) (Table 5)

**Table 5:** Associations between aspirin using and central nervous system tumor risk in subgroup meta-analyses

Studies groups	No of reports	Relative risk (95% CI)	Heterogeneity		*P* for test
			*P* value	I^2^ (%)	
Total	11	0.88 (0.79–0.95)	0.014	54.8%	0.006
**Tumour subtype**					
Glioma	8	0.86 (0.76–0.95)	0.013	43.8%	< 0.001
Meningioma	1	0.85 (0.65–1.12)			0.253
Glioblastoma	2	0.91 (0.55–1.50)	0.038	76.7%	0.701
**Study design**					
Case–control	8	0.83 (0.72–0.96)	0.032	54.4%	0.001
Cohort	3	0.92 (0.82–0.96)	0.055	65.5%	0.025
**No of participants**					
≥ 10 000	10	0.85 (0.75–0.96)	0.021	54.1%	0.003
< 10 000	1	1.08 (0.64–1.34)			0.651
**No of cases**					
≥ 1000	10	0.85 (0.75–0.96)	0.021	54.1%	0.003
< 1000	1	1.08 (0.64–1.34)			0.651
**Study quality**					
Score ≥ 7	10	0.85 (0.75–0.96)	0.021	54.1%	0.003
Score < 7	1	1.08 (0.64–1.34)			0.651

### Dose-response between NSAIDs use and CNS tumors risk

**Figure 2:**
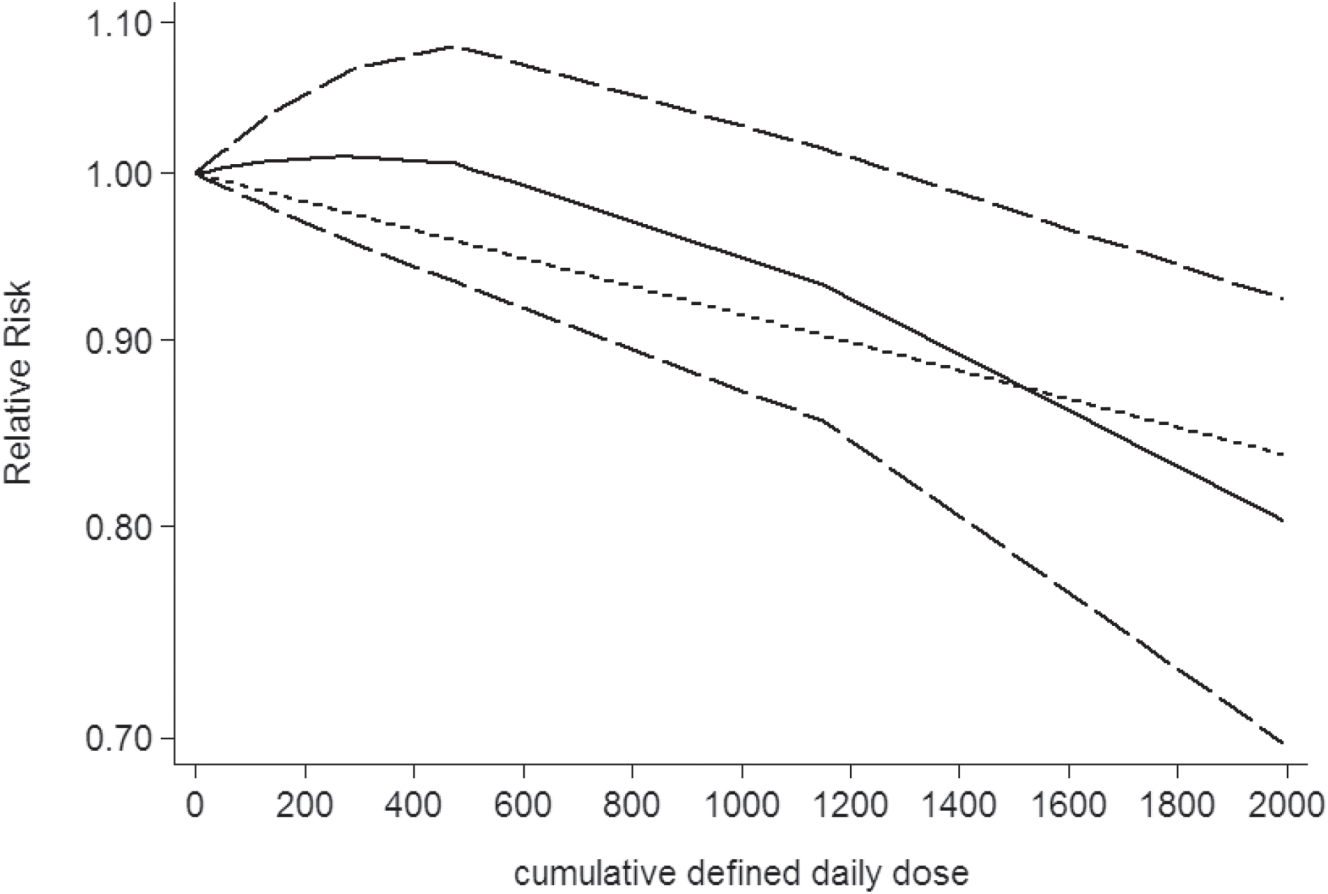
Dose-response relationship between cumulative daily dose of NSAIDs use in relation to risk of central nervous system tumor

**Figure 3:**
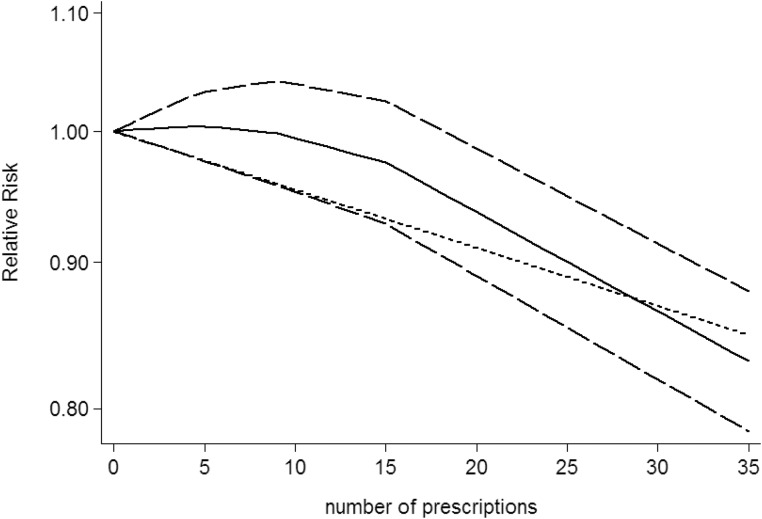
Dose-response relationship between number of prescriptions of NSAIDs use in relation to risk of central nervous system tumor

**Figure 4:**
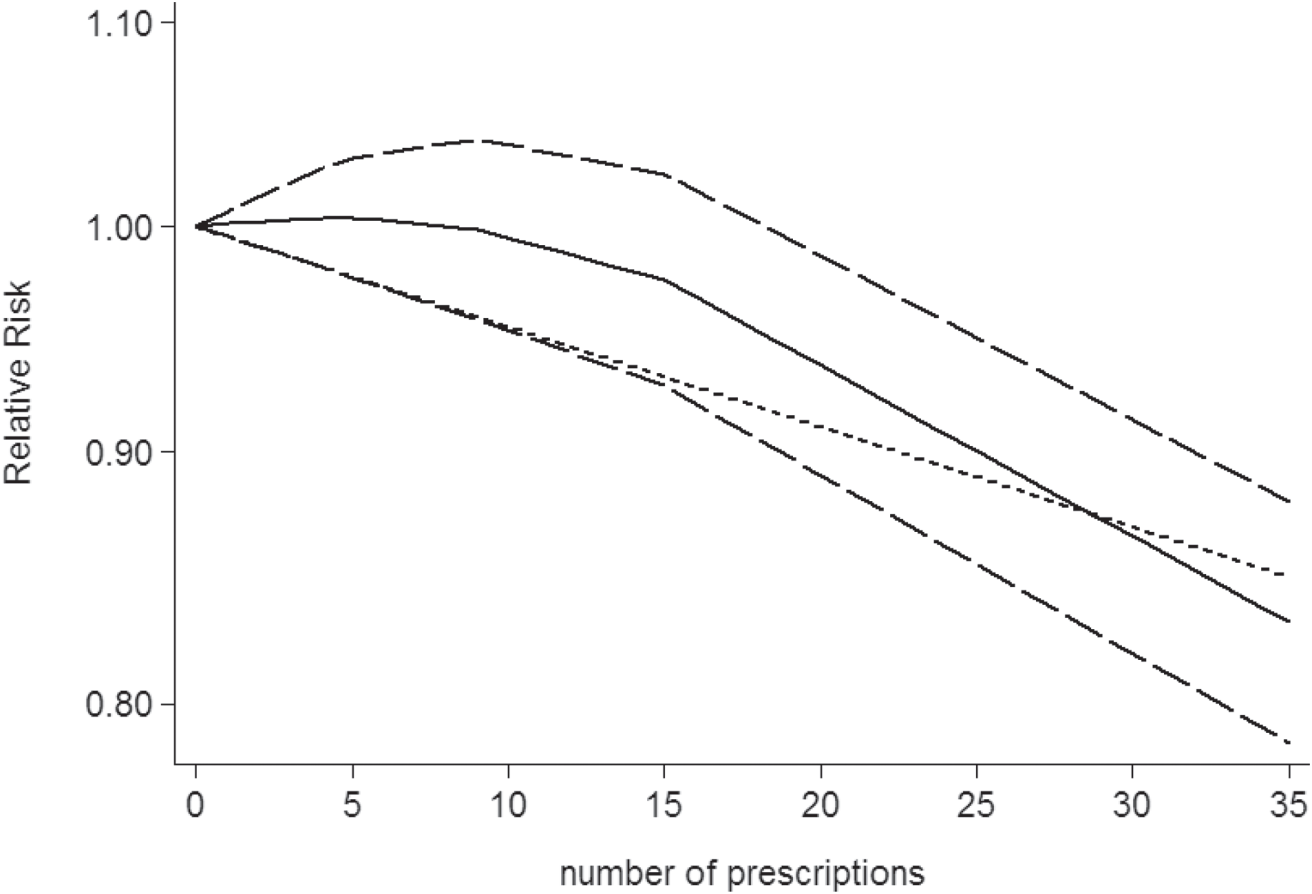
Dose-response relationship between number of prescriptions of non-aspirin NSAIDs use in relation to risk of central nervous system tumor

**Figure 5:**
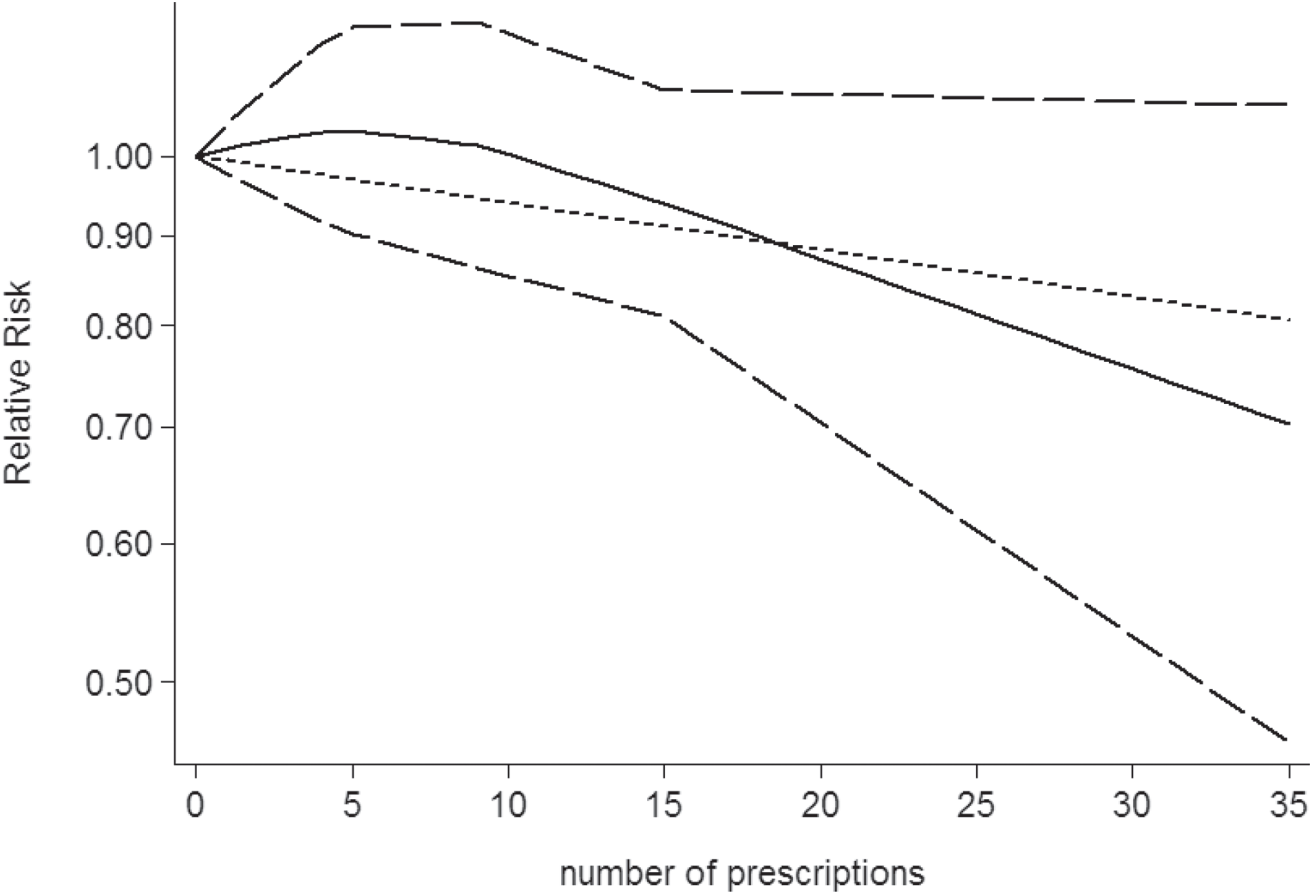
Dose-response relationship between number of prescriptions of aspirin use in relation to risk of central nervous system tumor

**Figure 6:**
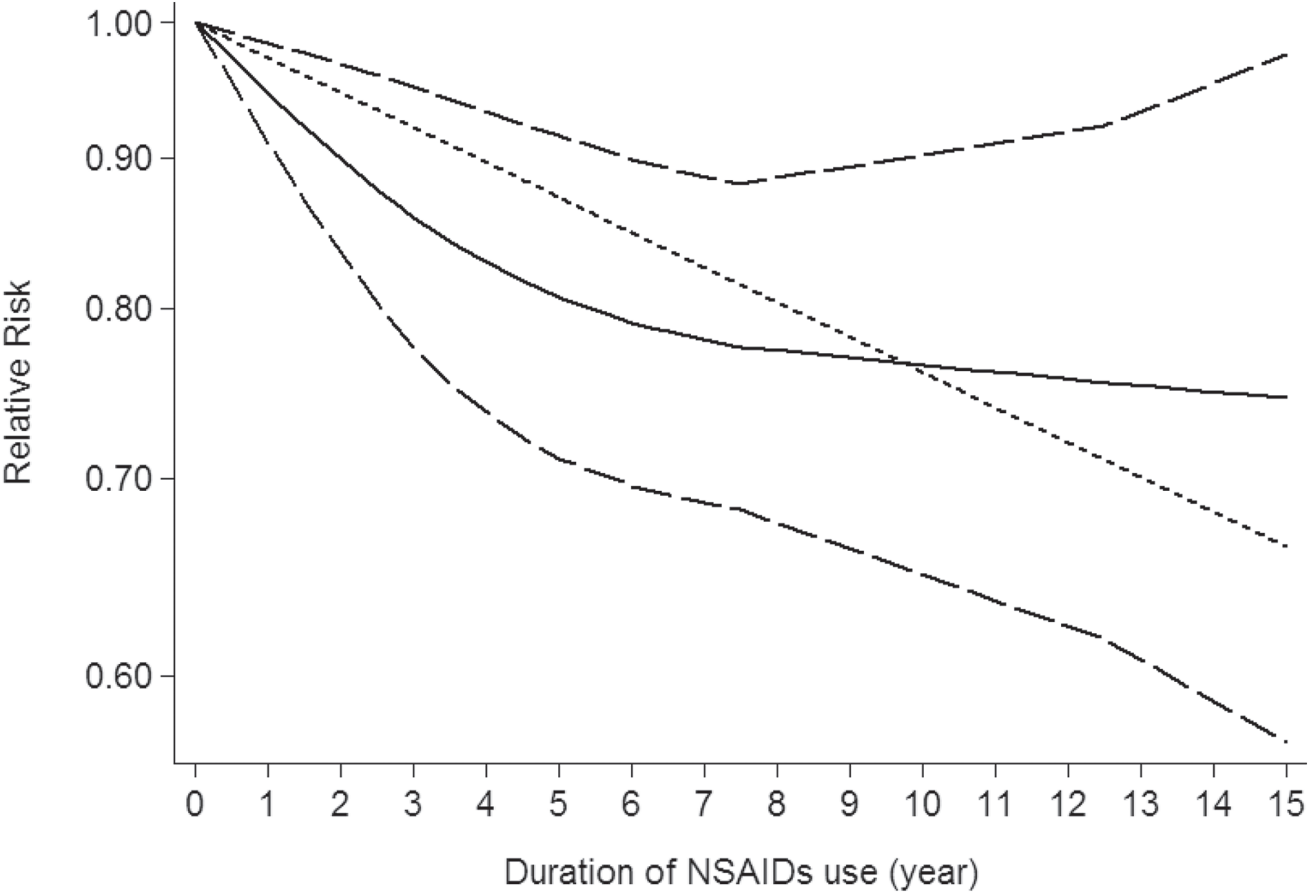
Dose-response relationship between duration of NSAIDs use in relation to risk of central nervous system tumor

**Figure 7:**
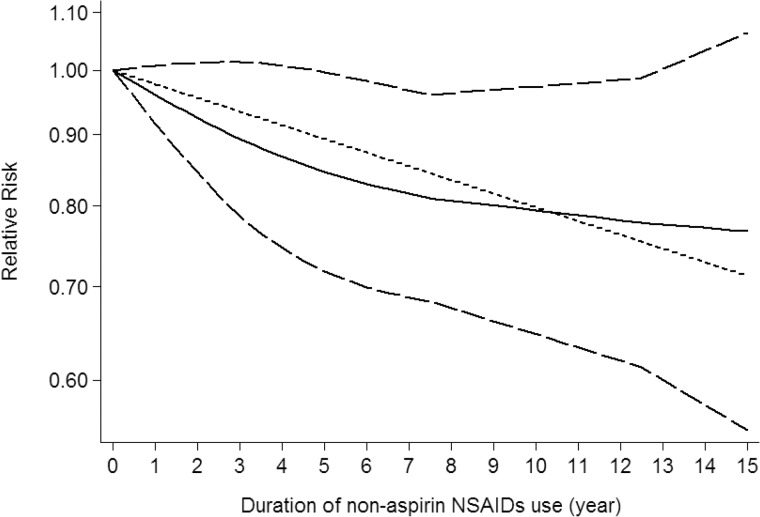
Dose-response relationship between duration of non-aspirin NSAIDs use in relation to risk of central nervous system tumor

**Figure 8:**
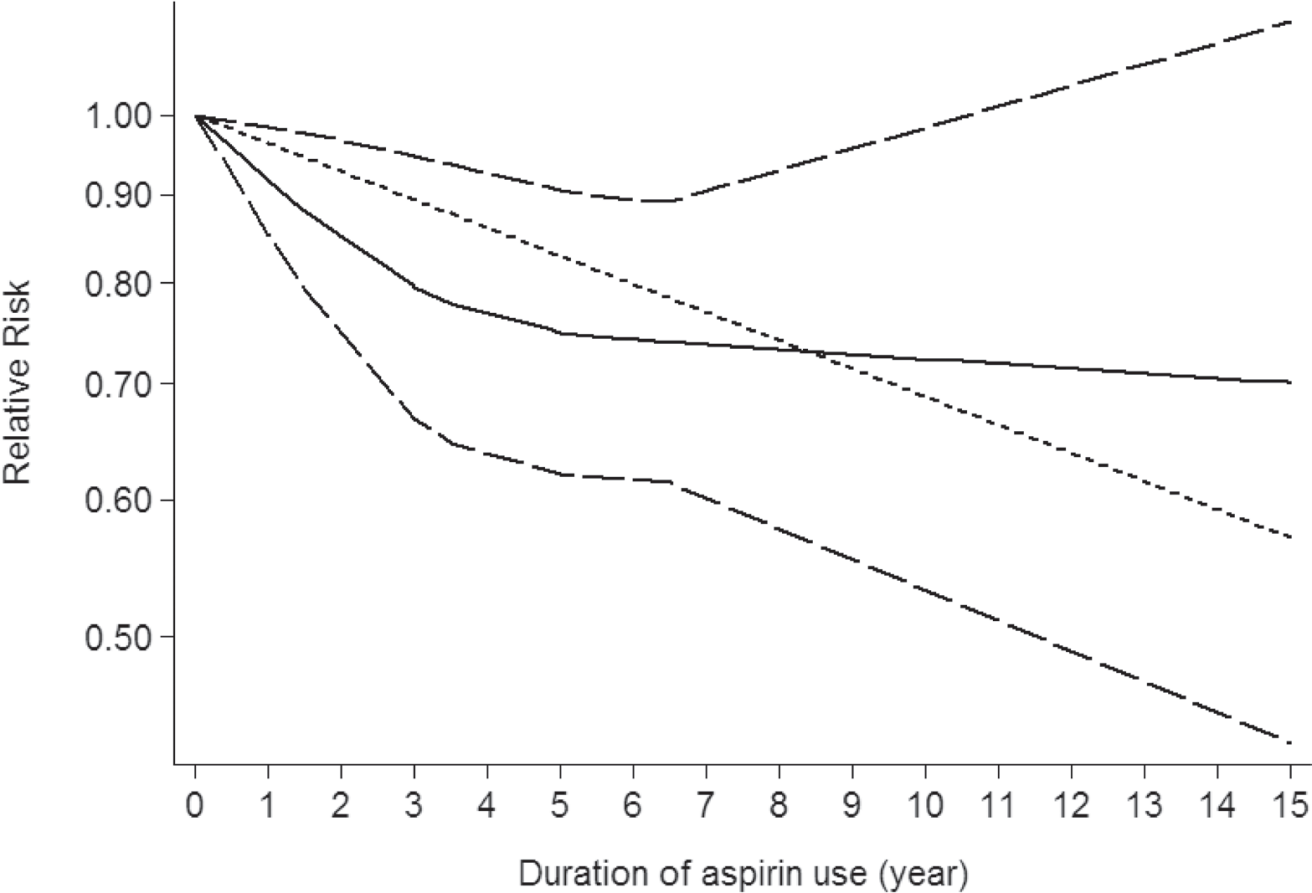
Dose-response relationship between duration of aspirin use in relation to risk of central nervous system tumor

Use restricted cubic spline function, the test for a nonlinear dose-response relationship was significant (likelihood ratio test, *P* < 0.001), suggesting curvature in the relationship between NSAIDs use and CNS tumors risk. Increasing cumulative 100 defined daily dose of NSAIDs use was associated with a 5% decrement of CNS tumors risk, the summary relative risk of CNS tumors risk for an per cumulative 100 defined daily dose of NSAIDs use was 0.95 (95% CI: 0.92–0.98, *P* = 0.003) (Figure 2). Furthermore, increasing NSAIDs use (per 3 prescriptions increment) was associated with a 7% decrement of CNS tumors risk, the summary relative risk of CNS tumors risk for an per 3 prescriptions increment of NSAIDs use was 0.93 (95% CI: 0.88–0.97, *P* < 0.001) (Figure 3). Increasing non-aspirin NSAIDs use (per 3 prescriptions increment) was associated with a 7% decrement of CNS tumors risk, the summary relative risk of CNS tumors risk for an per 3 prescriptions increment of non-aspirin NSAIDs use was 0.93 (95% CI: 0.89–0.97, *P* < 0.001) (Figure 4). Increasing aspirin use (per 3 prescriptions increment) was associated with a 10% decrement of CNS tumors risk, the summary relative risk of CNS tumors risk for an per 3 prescriptions increment of aspirin use was 0.90 (95% CI: 0.85–0.95, *P* < 0.001) (Figure 5). Additionally, increasing per 2 year of duration of NSAIDs use was associated with a 6% decrement of CNS tumors risk, the summary relative risk of CNS tumors risk for an per 2 year of duration of NSAIDs use was 0.94 (95% CI: 0.92–0.98, *P* = 0.001) (Figure 6), increasing per 2 year of duration of non-aspirin NSAIDs use was associated with a 8% decrement of CNS tumors risk, the summary relative risk of CNS tumors risk for an per 2 year of duration of non-aspirin NSAIDs use was 0.92 (95% CI: 0.87–0.97, *P* = 0.001) (Figure 7), increasing per 2 year of duration of aspirin use was associated with a 6% decrement of CNS tumors risk, the summary relative risk of CNS tumors risk for an per 2 year of duration of aspirin use was 0.94 (95% CI: 0.88–0.98, *P* = 0.005) (Figure 8).

### Subgroup analyses

Subgroup analysis was performed to check the stability of the primary outcome. Subgroup meta-analyses in study design, study quality, number of participants and number of cases showed consistent findings (Tables 3–5).

### Sensitivity analysis

Sensitivity analysis was conducted to assess the stability of the results. The results show the association between NSAIDs use and CNS tumors risk were stable (Supplementary Figures 1–3).

### Publication bias

Publication bias of NSAIDs use was evaluated with both Begg's and Egger's tests. Results from Egger's tests indicated no evidence of publication bias among these studies (Supplementary Table 1). A funnel plot for publication bias assessment is illustrated in Supplementary Figures 4–6.

## DISCUSSION

In the current meta-analysis was based on 12 case-control or cohort study, with 667085 participants with 19394 incident cases. Thus, this meta analysis provides the most up-to-date epidemiological evidence supporting NSAIDs use is helpful for CNS tumors. A dose-response analysis revealed that increasing cumulative 100 defined daily dose of NSAIDs use was associated with a 5% decrement of CNS tumors risk, increasing NSAIDs or non-aspirin NSAIDs or aspirin use (per 3 prescriptions increment) was associated with a 7%, 7%, 10% decrement of CNS tumors risk, increasing per 2 year of duration of NSAIDs or non-aspirin NSAIDs or aspirin use was associated with a 6%, 8%, 6% decrement of CNS tumors risk.

Previous meta-analysis reported the association between NSAIDs use and brain tumour risk, and found NSAIDs use did not appear to be associated with brain tumour risk [24]. Though, the result is quite different with ours, it still exist some problems. On the one hand, previous meta-analysis included just ten reports from ten studies, our meta-analysis included thirty-three independent reports from twelve studies, the quantity of reports involving in their meta-analysis was smaller, which weak the persuasive power of their research, and our results become more convincing. On the other hand, previous meta-analysis did not include all NSAIDs in the study, this may be affect the result and all NSAIDs acted as a independent report in our meta-analysis.

Several plausible pathways may reasonable for the relationship between NSAIDs use and CNS tumors risk. Firstly, NSAIDs have been found to inhibit or kill glioma cells, and the nonsteroidal anti-inflammatory drug can suppresses the growth and induces apoptosis of human glioblastoma cells via the NF-kappaB pathway [25–27]. Secondly, refractoriness of glioblastoma multiforme (GBM) largely depends on its radioresistance, and celecoxib enhances radiosensitivity of hypoxic glioblastoma cells through endoplasmic reticulum stress [28–31]. Third, NSAIDs use can significantly enhance glioblastoma radiosensitivity, reduced clonogenic survival, and prolonged survival of glioblastoma-implanted mice by inhibition of tumor angiogenesis with extensive tumor necrosis and reduce angiogenesis [32–34]. Fourth, NSAIDs use is sufficient to render unmodified tumor cells immunogenic in immunotherapy of experimental brain tumors, and stimulate anti-tumour immune reactions *in vitro* and in established animal models [35–37]. However, the potential mechanisms of NSAIDs use and tumor growth still remain unclear and controversial.

To our knowledge, this is the first comprehensive study to identify and quantify the potential dose-response association between NSAIDs use and CNS tumors risk in both men and women. Although, we performed this meta-analysis very carefully, some limitations must be considered in the current meta-analysis. Firstly, despite we searched all studies describing the association between NSAIDs use and CNS tumors risk, there are only 12 studies about NSAIDs use and CNS tumors risk, the number of eligible studies was still limited. On the other hand, 12 studies from only three countries, different ethnic population are warranted to validate the association between NSAIDs use and CNS tumors risk. Thirdly, we only select literature that written by English, which may have resulted in a language or cultural bias, other languages should be chosen in the further. Fourth, in the subgroup analysis in CNS tumors type, there has no insufficient statistical power to check a dose-response in different CNS tumors type, large data in different CNS tumors type is warranted to validate this association.

In conclusion, our findings underscore the notion that NSAIDs use was significantly associated with CNS tumors risk decrement. In the future, large-scale case-control and population based association studies must be performed in the future to validate the risk identified in the current meta-analysis.

## MATERIALS AND METHODS

Our meta-analysis was conducted according to the Meta-analysis Of Observational Studies in Epidemiology (MOOSE) checklist [38].

### Search strategy

We included eligible studies investigating the relationship of NSAIDs use and CNS tumors risk. To develop a flexible, non-linear, r meta-regression model, we required that an eligible study should have categorized into 3 or more levels.

Eligible studies were systematically searched of PubMed and Embase update to July 2017 for case control or cohort studies examining the relationship between NSAIDs use and CNS tumors risk, with keywords including “brain cancer” [MeSH] OR “glioma”[MeSH] OR “glioblastoma” [MeSH] OR “meningioma” [MeSH] AND “aspirin” [MeSH] OR “NSAIDs” [MeSH] OR “ibuprofen”[MeSH] OR “naproxen” [MeSH] OR “indomethacin” [MeSH] OR “meloxicam” [MeSH] OR “nimesulide” [MeSH] OR “celecoxib” [MeSH] OR “rofecoxib”[MeSH] OR “acetaminophen” [MeSH] OR “diclofenac”[MeSH]. We refer to the relevant original essays and commentary articles to determine further relevant research.

### Study selection

Two independent researchers investigate information the correlation between NSAIDs use and CNS tumors risk: outcome was CNS tumors; the relative risks at least three quantitative categories of NSAIDs use and CNS tumors risk. Moreover, we precluded non-human studies, reviews, editorials and published letters.

### Data extraction

Use standardized data collection tables to extract data. We extracted the following information: first author; publication year; age; country; sex; cases and participants; the categories of NSAIDs use; relative risk(RR) or odds ratio (OR). We collect the risk estimates with multivariable-adjusted. According to the Newcastle-Ottawa scale [39], quality assessment was performed for non-randomized studies.

### Statistical analysis

We pooled relative risk estimates to measure the association between NSAIDs use and CNS tumors risk; the hazard ratio were considered equivalent to the relative risk [40]. Results in different subgroups of NSAIDs use and CNS tumors risk were treated as two separate reports.

Due to different definitions cut-off points in the included studies for categories, we performed a relative risk estimates by increasing cumulative 100 defined daily dose of NSAIDs use or per 3 prescriptions of NSAIDs use or per 2 year of duration of NSAIDs use the method recommended by Greenland, Longnecker and Orsini and colleagues [41]. In addition, use restricted cubic splines to evaluate the non-linear association between NSAIDs use and CNS tumors risk, with three knots at the 10th, 50th, and 90th percentiles of the distribution. A flexible meta-regression based on restricted cubic spline (RCS) function was used to fit the potential non-linear trend, and generalized least-square method was used to estimate the parameters. This procedure treats NSAIDs use (continuous data) as an independent variable and logRR of diseases as a dependent variable, with both tails of the curve restricted to linear. A *P* value is calculated for linear or non-linear by testing the null hypothesis that the coefficient of the second spline is equal to zero [42].

We use STATA software 12.0 (STATA Corp, College Station, TX, USA) to evaluate the relationships between NSAIDs use and CNS tumors risk. Heterogeneity among studies used Q test and I^2^ statistic to assess. If *P*_Q_< 0.10 or I^2^ > 50%, random-effect model was chosen, otherwise, fixed-effect mode was applied. Begg's and Egger's tests were to assess the publication bias of each study. *P* < 0.05 was considered signifcant for all tests.

## SUPPLEMENTARY MATERIALS FIGURES AND TABLES


