# Supplementary material for: Association between nonsteroidal anti-inflammatory drugs use and risk of central nervous system tumors: a dose-response meta analysis
Source: Oncotarget. 2017 Oct 11;8(60):102486–98. doi: 10.18632/oncotarget.21829 (PMC5731974; doi:10.18632/oncotarget.21829)
Supplement: Supplementary file 1 [file oncotarget-08-102486-s001.pdf]

Association between nonsteroidal anti-inflammatory drugs use and risk of central nervous system tumors: a dose-response meta analysis

SUPPLEMENTARY MATERIALS

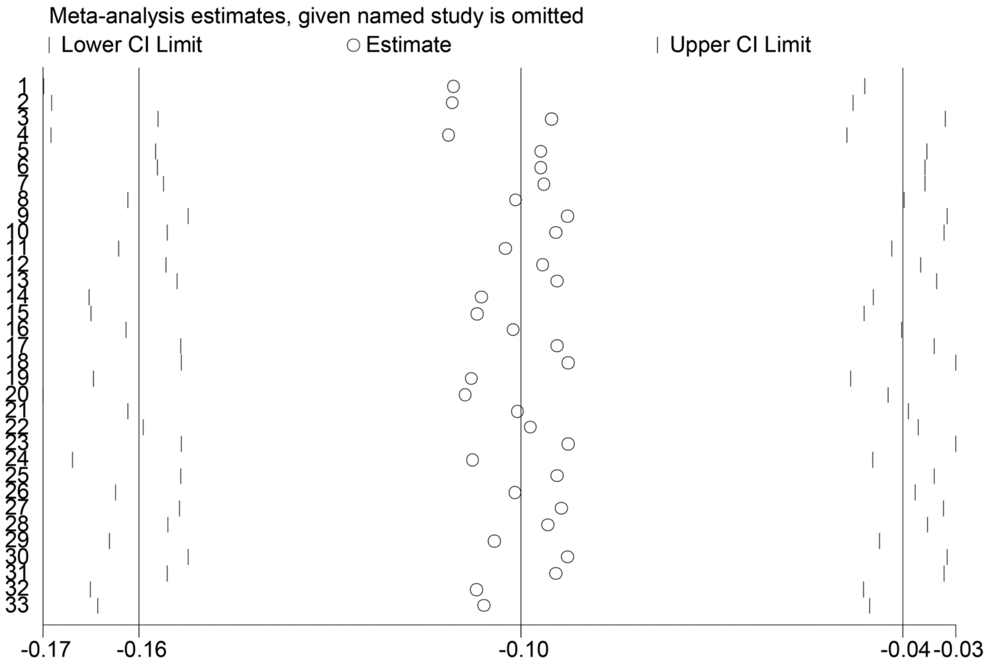

Supplementary Figure 1: Forest plots of sensitivity analysis for the meta-analysisn of NSAIDs use and CNS tumors risk.

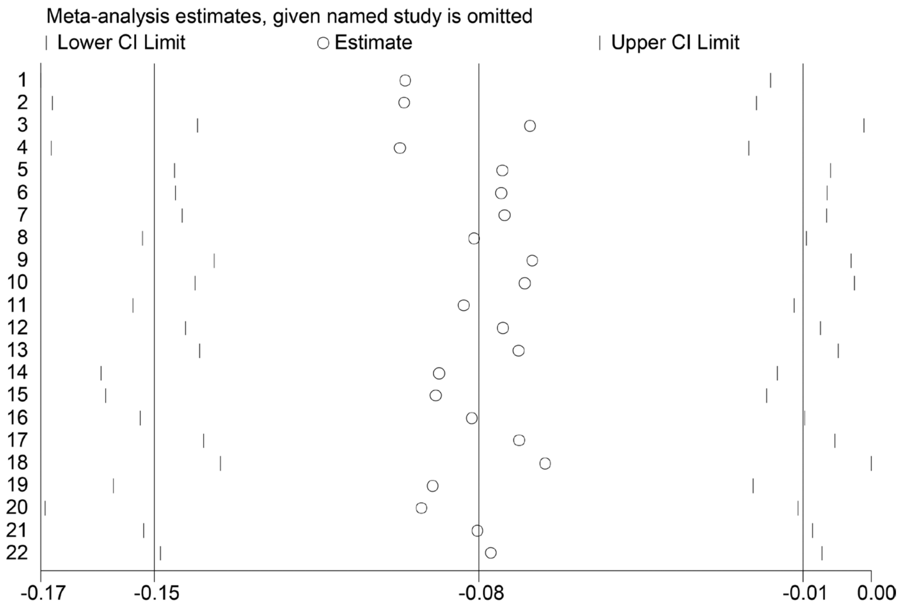

Supplementary Figure 2: Forest plots of sensitivity analysis for the meta-analysisn of non-aspirin NSAIDs use and CNS tumors risk.

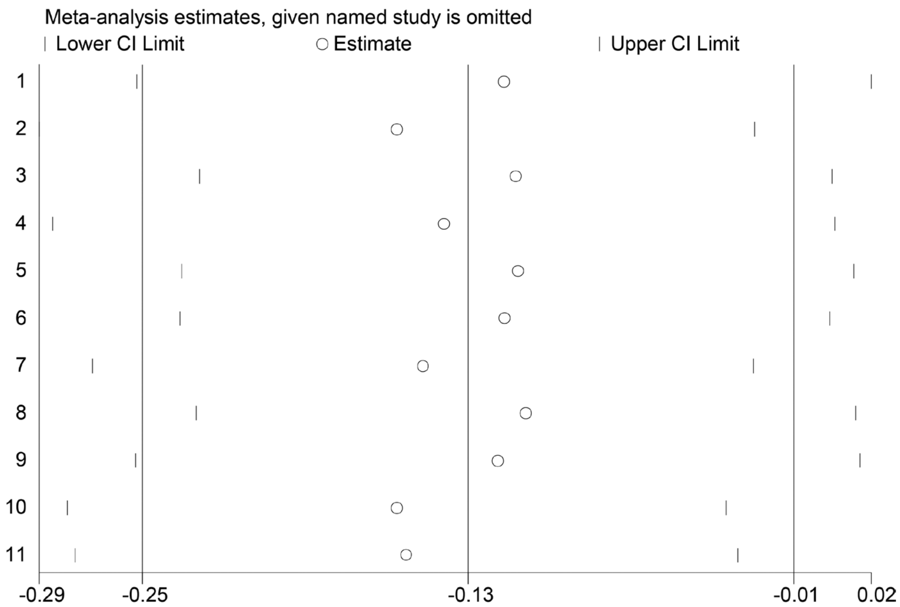

Supplementary Figure 3: Forest plots of sensitivity analysis for the meta-analysisn of aspirin use and CNS tumors risk.

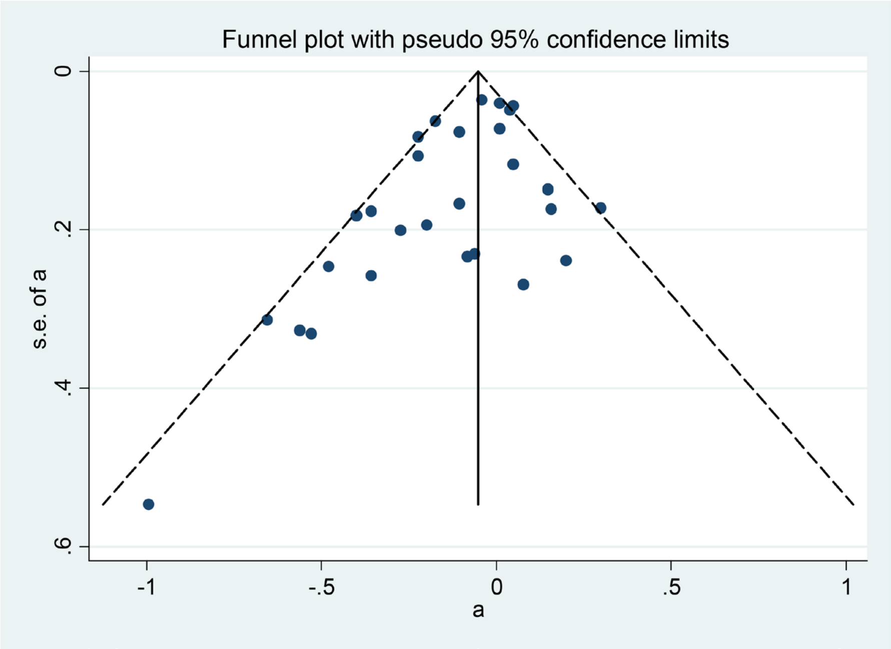

Supplementary Figure 4: A funnel plot for the meta-analysis of NSAIDs use and CNS tumors risk.

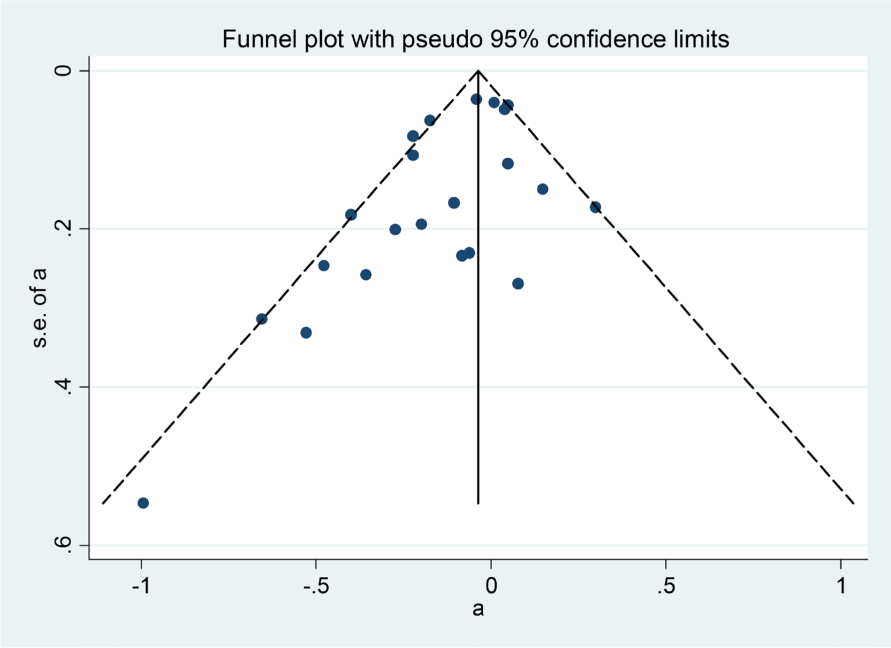

Supplementary Figure 5: A funnel plot for the meta-analysis of non-aspirin NSAIDs use and CNS tumors risk.

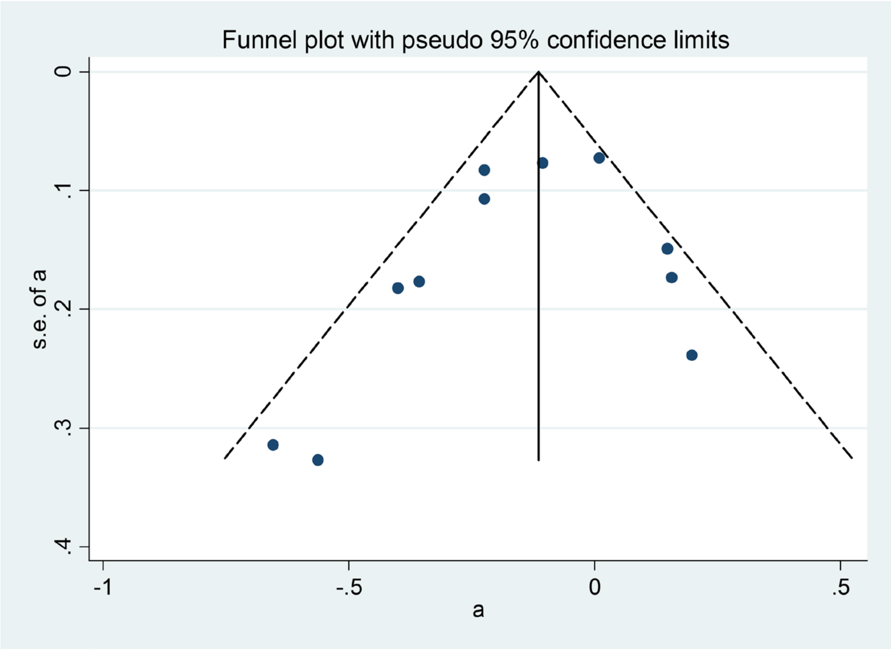

Supplementary Figure 6: A funnel plot for the meta-analysis of aspirin use and CNS tumors risk.

Supplementary Table 1: Publication bias analysis of the meta-analysis

	Test	t	95% CI	*P*
**NSAIDs use**	Begg's test			0.128
	Egger's test	1.12	−0.29, 1.03	0.267
**Non-aspirin NSAIDs use**	Begg's test			0.929
	Egger's test	1.27	−2.98, 2.86	1.000
**Aspirin use**	Begg's test			0.118
	Egger's test	0.89	−1.26, 2.78	0.401
